# Prognostic Impact of Combined Contrast-Induced Acute Kidney Injury and Hypoxic Liver Injury in Patients with ST Elevation Myocardial Infarction Undergoing Primary Percutaneous Coronary Intervention: Results from INTERSTELLAR Registry

**DOI:** 10.1371/journal.pone.0159416

**Published:** 2016-07-14

**Authors:** Sang-Don Park, Jeonggeun Moon, Sung Woo Kwon, Young Ju Suh, Tae-Hoon Kim, Ho-Jun Jang, Jon Suh, Hyun Woo Park, Pyung Chun Oh, Sung-Hee Shin, Seong-Il Woo, Dae-Hyeok Kim, Jun Kwan, WoongChol Kang

**Affiliations:** 1 Department of Cardiology, Inha University Hospital, Incheon, Republic of Korea; 2 Department of Cardiology, Gil Medical Center, Gachon University, Incheon, Republic of Korea; 3 Department of Biomedical Sciences, Inha University School of Medicine, Incheon, Republic of Korea; 4 Department of Cardiology, Sejong General Hospital, Bucheon, Republic of Korea; 5 Department of Cardiology, Soon Chun Hyang University Bucheon Hospital, Bucheon, Republic of Korea; University Hospital Medical Centre, GERMANY

## Abstract

**Background:**

Besides contrast-induced acute kidney injury(CI-AKI), adscititious vital organ damage such as hypoxic liver injury(HLI) may affect the survival in patients with ST-elevation myocardial infarction (STEMI). We sought to evaluate the prognostic impact of CI-AKI and HLI in STEMI patients who underwent primary percutaneous coronary intervention (PCI).

**Methods:**

A total of 668 consecutive patients (77.2% male, mean age 61.3±13.3 years) from the INTERSTELLAR STEMI registry who underwent primary PCI were analyzed. CI-AKI was defined as an increase of ≥0.5 mg/dL in serum creatinine level or 25% relative increase, within 48h after the index procedure. HLI was defined as ≥2-fold increase in serum aspartate transaminase above the upper normal limit on admission. Patients were divided into four groups according to their CI-AKI and HLI states. Major adverse cardiovascular and cerebrovascular events (MACCE) defined as a composite of all-cause mortality, non-fatal MI, non-fatal stroke, ischemia-driven target lesion revascularization and target vessel revascularization were recorded.

**Results:**

Over a mean follow-up period of 2.2±1.6 years, 94 MACCEs occurred with an event rate of 14.1%. The rates of MACCE and all-cause mortality were 9.7% and 5.2%, respectively, in the no organ damage group; 21.3% and 21.3% in CI-AKI group; 18.5% and 14.6% in HLI group; and 57.7% and 50.0% in combined CI-AKI and HLI group. Survival probability plots of composite MACCE and all-cause mortality revealed that the combined CI-AKI and HLI group was associated with the worst prognosis (p<0.0001 for both).

**Conclusion:**

Combined CI-AKI after index procedure and HLI on admission is associated with poor clinical outcomes in patients with STEMI who underwent primary PCI. **(INTERSTELLAR ClinicalTrials.gov number, NCT02800421.)**

## Introduction

Even after successful primary percutaneous coronary intervention (PCI), both the short and long term prognoses of patients with ST-elevation myocardial infarction (STEMI) are considerably poor [1,2]. Conventional risk factors, including diabetes mellitus, are known to be associated with worse outcomes in these patients [3]. Left ventricular ejection fraction (LVEF) and Killip class on admission, as well as multi-vessel disease (MVD) are also well-known parameters suspected of contributing to poorer prognosis [4,5]. Since adscititious vital organ damage may affect the survival of patients with STEMI, previous studies have revealed the importance of contrast-induced acute kidney injury (CI-AKI) in interventionally-treated patients with STEMI, in terms of prognosis [6–9]. A recent study had also proposed the prognostic impact of hypoxic liver injury (HLI) among patients with STEMI [10]. However, there is lack of data regarding the prognostic value of combined CI-AKI and HLI among patients with STEMI. Therefore, we sought to evaluate the prognostic impact of CI-AKI and HLI in patients with STEMI who underwent primary PCI.

## Materials and Methods

### Study design and patient selection

This was a multi-center study that consisted of 4 hospitals (Inha University Hospital, Gachon University Gil Medical Center, Sejong General Hospital, and Soon Chun Hyang University Bucheon Hospital) in the Incheon-Bucheon province. These 4 hospital established a STEMI registry designated as INTERSTELLAR (**IN**cheon-Bucheon cohor**T** of patients who und**ER**went primary PCI for acute **ST-El**evation myocardial inf**AR**ction) [11]. From the INTERSTELLAR registry cohort, a total of 668 consecutive patients (77.2% male, mean age 61.3±13.3 years) with STEMI who underwent primary PCI between 2007 and 2014 were enrolled. The study protocol was approved by the Institutional Review Board of the Inha University Hospital, Inha University College of Medicine (INHAUH 2016-05-015), and written consent was obtained from each patient.

Primary PCI was performed according to standard clinical practice [12]. Pharmacological therapy, temporary pacemaker insertion, and intra-aortic balloon pump support were left to the operators’ discretion. Patients were divided into four groups according to their CI-AKI and HLI states: no organ damage, CI-AKI only, HLI only, combined CI-AKI and HLI. The baseline risk factors, coronary angiographic findings, length of follow-up, and major adverse cardiovascular and cerebrovascular event (MACCE), which include all-cause mortality and non-fatal myocardial infarction (MI), non-fatal stroke, ischemia-driven target lesion revascularization (TLR) and target vessel revascularization (TVR) were recorded. This study is registered on ClinicalTrials.gov under the identifier NCT02800421.

### Definition of variables and measurements

Hypertension was defined as systolic blood pressure of ≥140 mmHg, diastolic blood pressure of ≥90 mmHg, or by antihypertensive prescription. Type 2 diabetes mellitus was defined by oral hypoglycemic agents or insulin prescription, fasting plasma glucose concentration of ≥126 mg/dL, glycosylated hemoglobin (HbA1c) concentration of ≥6.5%, or known but untreated hyperglycemia. Dyslipidemia was defined by total cholesterol level of ≥240 mg/dL, LDL cholesterol level of ≥130 mg/dL, HDL cholesterol level of <40 mg/dL, triglyceride level of ≥200 mg/dL, and/or by lipid-lowering prescription. STEMI was defined as a typical chest pain lasting for >30 min within the last 24h, with electrocardiographic findings of ST-elevation >1 mm in at least two consecutive leads or new-onset left bundle branch block, and 2-fold elevation of serum levels of troponin-I or creatine kinase-MB above the upper normal limit. Obstructive CAD was defined as ≥50% luminal narrowing and the extent of obstructive CAD was categorized according to the number of vessels involved (1, 2, or 3) [13]. CI-AKI was defined as an increase of ≥0.5 mg/dL in serum creatinine level or a 25% relative increase, within 48h after the index procedure [7]. HLI was defined as ≥2-fold increase of serum aspartate transaminase level above upper normal limit on admission [10].

### Endpoint determination and follow-up data acquisition

The primary endpoint was all-cause mortality, while the secondary endpoint was composite major adverse cardiovascular and cerebrovascular event (MACCE), defined as all-cause mortality, non-fatal MI, non-fatal stroke, and ischemia-driven TLR/TVR,during the follow-up period [14,15]. For patients with multiple cardiovascular and cerebrovascular events, only the first event was considered for analysis. Patient follow-up data were collected using electronic medical record review and/or standardized telephone interviews.

### Data analysis and statistical methods

Continuous data were expressed as a mean value ± standard deviation and/or median value (interquartile range), as appropriate. Categorical data were presented as a percentage or absolute number. Analyses of continuous data were performed using the analysis of variance (ANOVA) test or Kruskal-Wallis test, where appropriate. Fisher’s exact test with adjustments by permutation resampling were performed to assess differences of mortality rates among the four groups [16]. Cumulative event rates, as a function over time, were estimated using the Kaplan-Meier method, and survival curves of all-cause mortality and MACCE were compared using the log-rank test. Cox proportional hazards regression analyses were performed to determine the significant risk factors for all-cause mortality and MACCE such as clinical characteristics, laboratory parameters, Killip classification, LVEF, MVD, CI-AKI, and HLI. Hazard ratios (HR) were calculated as an estimate of the risk associated with a particular variable, with 95% confidence intervals (CI). All analyses were performed using SPSS version 19.0 (SPSS, Chicago, IL, USA) and SAS version 9.3 (SAS Institute, Cary, NC, USA). A p-value of less than 0.05 was considered statistically significant.

## Results

### Baseline characteristics of the study population and comparison among patients according to CI-AKI and HLI states

Of the 668 patients, 465 patients (69.6%) were allocated to the no organ damage group, 47 patients (7.0%) to the CI-AKI only group, 130 patients (19.5%) to the HLI only group, and 26 patients (3.9%)to the combined CI-AKI and HLI group. A summary of the baseline, laboratory, and angiographic characteristics according to CI-AKI and HLI state is shown in Table 1. Patients with combined CI-AKI and HLI tended to be older (p = 0.029) and hypertensive (p = 0.039). More patients with combined CI-AKI and HLI had faster heart rate (p<0.0001), and worse Killip class during their initial clinical presentation (p = 0.001). The baseline creatinine level was higher (p<0.0001), while LVEF was lower (p<0.0001) in the combined CI-AKI and HLI group. In addition, MVD was more frequent in patients with CI-AKI than in those without CI-AKI (p = 0.018). Intra-aortic balloon pump support was used more frequently in the combined CI-AKI and HLI group (11.5%). However, there was no statistical difference among the groups (p = 0.349) (Table 1).

**Table 1:** Baseline, laboratory, and angiographic characteristics according to CI-AKI and HLI states.

Variable	Total(n = 668)	No organ damage (n = 465)	CI-AKI only(n = 47)	HLI only(n = 130)	Combined CI-AKI and HLI (n = 26)	p-value
Age (years)	61.3±13.3	60.9±13.0	63.6±14.3	60.6±13.5	68.2±14.7	0.029
	61.0 (51.0–71.0)	61.0 (51.0–70.0)	62.0 (53.0–75.0)	60.0 (49.8–71.0)	69.5 (59.8–76.5)	0.024
Male gender (%)	516 (77.2%)	361 (77.6%)	35 (74.5%)	100 (76.9%)	20 (76.9%)	0.968
Diabetes (%)	201 (30.1%)	144 (31.0%)	18 (38.3%)	32 (24.6%)	7 (26.9%)	0.302
Hypertension (%)	345 (51.6%)	237 (51.0%)	29 (61.7%)	60 (46.2%)	19 (73.1%)	0.039
Dyslipidemia (%)	165 (24.7%)	110 (23.7%)	16 (34.0%)	34 (26.2%)	5 (19.2%)	0.385
SBP (mmHg)	123.2±27.2	123.2±26.9	130.0±28.5	119.9±27.6	127.6±27.6	0.142
	120.0 (108.0–140.0)	120.0 (107.0–140.0)	128.5 (119.5–143.0)	120.0 (100.0–136.8)	130.0 (110.0–146.5)	0.159
DBP (mmHg)	75.9±18.1	75.9±17.7	80.4±16.7	74.5±20.1	75.7±16.6	0.304
	79.0 (63.0–84.0)	77.0 (61.0–85.0)	80.0 (70.8–90.5)	80.0 (60.0–83.0)	75.0 (66.5–80.0)	0.169
Heart rate (bpm)	78.0±21.0	76.3±20.4	82.9±21.4	79.4±20.7	93.0±26.3	<0.0001
	77.0 (65.0–88.0)	76.0 (64.0–85.0)	78.5 (69.5–92.0)	78.0 (66.0–90.0)	87.0 (79.8–110.5)	<0.0001
Killip class (%)						0.001
1	495 (74.1%)	353 (79.5%)	33 (70.2%)	98 (77.2%)	11 (44.0%)	
2	38 (5.7%)	24 (5.4%)	6 (12.8%)	4 (3.1%)	4 (16.0%)	
3	55 (8.2%)	31 (7.0%)	5 (10.6%)	12 (9.4%)	7 (28.0%)	
4	55 (8.2%)	36 (8.1%)	3 (6.4%)	13 (10.2%)	3 (12.0%)	
AST (mg/dL)	245.9±279.6	34.2±15.0	34.3±16.2	181.8±127.3	236.2±165.6	<0.0001
	36.0 (25.0–68.8)	30.0 (23.0–42.0)	29.0 (21.0–42.0)	141.5 (97.8–217.5)	173.0 (116.3–337.5)	<0.0001
ALT (mg/dL)	91.8±134.4	27.4±13.3	24.6±13.1	72.0±48.0	91.5±92.6	<0.0001
	28.0 (19.0–43.0)	24.0 (18.0–35.0)	20.0 (16.0–31.0)	63.5 (38.0–89.3)	59.0 (40.5–97.8)	<0.0001
HLI (%)	156 (23.4%)	0 (0%)	0 (0%)	130 (100%)	26 (100%)	<0.0001
Baseline creatinine (mg/dL)	1.06±0.64	0.99±0.56	1.40±0.81	0.99±0.32	1.97±1.48	<0.0001
	0.93 (0.78–1.12)	0.90 (0.77–1.06)	1.20 (0.79–1.77)	0.98 (0.80–1.13)	1.51 (1.00–2.49)	<0.0001
Peak creatinine (mg/dL)	1.22±0.91	1.07±0.41	2.13±2.30	1.21±0.74	2.34±1.72	<0.0001
	1.01 (0.89–1.21)	1.00 (0.86–1.16)	1.39 (1.00–2.47)	1.04 (0.90–1.21)	1.96 (1.06–2.94)	<0.0001
CI-AKI (%)	73 (10.9%)	0 (0%)	47 (100%)	0 (0%)	26 (100%)	<0.0001
Initial CK (U/L)	499.7±850.4	216.4±355.3	272.8±277.3	1393.6±1279.8	1497.7±1249.8	<0.0001
	161.0 (96.0–467.0)	129.0 (85.5–220.5)	174.0 (112.0–299.0)	1046.0 (480.0–1793.0)	886.0 (519.3–2247.0)	<0.0001
Initial CK-MB (μg/mL)	36.0±74.9	12.6±30.8	15.5±19.6	110.3±115.8	118.6±123.9	<0.0001
	6.5 (2.9–30.7)	4.3 (2.4–10.1)	7.2 (3.4–18.4)	80.5 (14.7–151.2)	88.2 (19.8–172.9)	<0.0001
Initial TnI (ng/mL)	4.82±21.19	1.56±14.54	1.99±7.62	15.20±35.11	13.04±24.89	<0.0001
	0.12 (0.03–0.93)	0.10 (0.01–0.27)	0.19 (0.04–0.83)	1.73 (0.41–7.80)	2.64 (0.98–8.50)	<0.0001
Peak CK (U/L)	1975.9±2807.6	1617.2±2498.7	1631.0±2772.5	3103.9±3339.6	3407.2±3391.7	<0.0001
	826.0 (172.0–2641.0)	484.0 (142.0–2099.0)	269.0 (156.0–1186.0)	1771.0 (1009.8–4258.8)	2522.0 (719.5–5188.3)	<0.0001
Peak CK-MB (μg/mL)	237.9±213.3	220.1±204.3	279.0±229.9	274.4±227.5	299.0±233.6	0.012
	172.3 (74.5–354.7)	160.3 (67.5–333.0)	276.3 (67.0–444.0)	220.3 (99.9–375.5)	264.7 (107.3–600.0)	0.014
Peak TnI (ng/mL)	53.65±95.97	48.98±96.04	42.07±84.25	68.41±96.87	80.00±102.74	0.088
	1.49 (0.07–69.00)	0.49 (0.02–54.75)	0.47 (0.05–43.15)	7.76 (1.25–109.90)	15.73 (1.09–176.43)	<0.0001
LVEF (%)	45.3±11.6	46.9±10.9	37.5±13.0	44.2±11.6	34.9±11.1	<0.0001
	45.0 (39.0–52.5)	46.0 (40.0–54.0)	38.0 (30.0–47.0)	43.0 (37.0–50.0)	34.0 (25.0–45.3)	<0.0001
CAD extent						0.018
1-vessel disease (%)	274 (41.0%)	199 (43.0%)	15 (31.9%)	51 (39.2%)	9 (34.6%)	
2-vessel disease (%)	214 (32.0%)	143 (30.9%)	10 (21.3%)	52 (40.0%)	9 (34.6%)	
3-vessel disease (%)	178 (26.6%)	121 (26.1%)	22 (46.8%)	27 (20.8%)	8 (30.8%)	
Multi-vessel disease (%)	392 (58.6%)	264 (57.0%)	32 (68.1%)	79 (60.8%)	17 (65.4%)	
Infarct-related artery						0.274
LAD (%)	344 (51.5%)	233 (50.3%)	24 (51.1%)	68 (52.3%)	19 (73.1%)	
LCX (%)	66 (9.9%)	52 (11.2%)	3 (6.4%)	10 (7.7%)	1 (3.8%)	
RCA (%)	247 (37.0%)	174 (37.6%)	19 (40.4%)	49 (37.7%)	5 (19.2%)	
LMCA (%)	9 (1.3%)	4 (0.9%)	1 (2.1%)	3 (2.3%)	1 (3.8%)	
IABP (%)	35 (5.2%)	23 (5.0%)	1 (2.1%)	8 (6.2%)	3 (11.5%)	0.349
Temporary pacemaker (%)	59 (8.8%)	44 (9.5%)	4 (8.5%)	11 (8.5%)	0 (0%)	0.426
30-day mortality (%)	38 (5.7%)	14 (3.0%)	3 (6.4%)	12 (9.2%)	9 (34.6%)	<0.0001

Categorical data are expressed as numbers (%) and continuous data are expressed as mean ± standard deviationand/or median (interquartile range). Abbreviations. AST = aspartate transaminase; ALT = alanine transaminase; CAD = coronary artery disease; CI-AKI = contrast induced acute kidney injury; CK = creatine kinase; CK-MB = creatine kinase-myocardial band; DBP = diastolic blood pressure; HLI = hypoxic liver injury; IABP = intra-aortic balloon pump; LAD = left anterior descending artery; LCX = left circumflex artery; LMCA = left main coronary artery; LVEF = left ventricular ejection fraction;RCA = right coronary artery; SBP = systolic blood pressure; TnI = troponin I.

### Thirty-day mortality according to CI-AKI and HLI states

**Fig 1:**
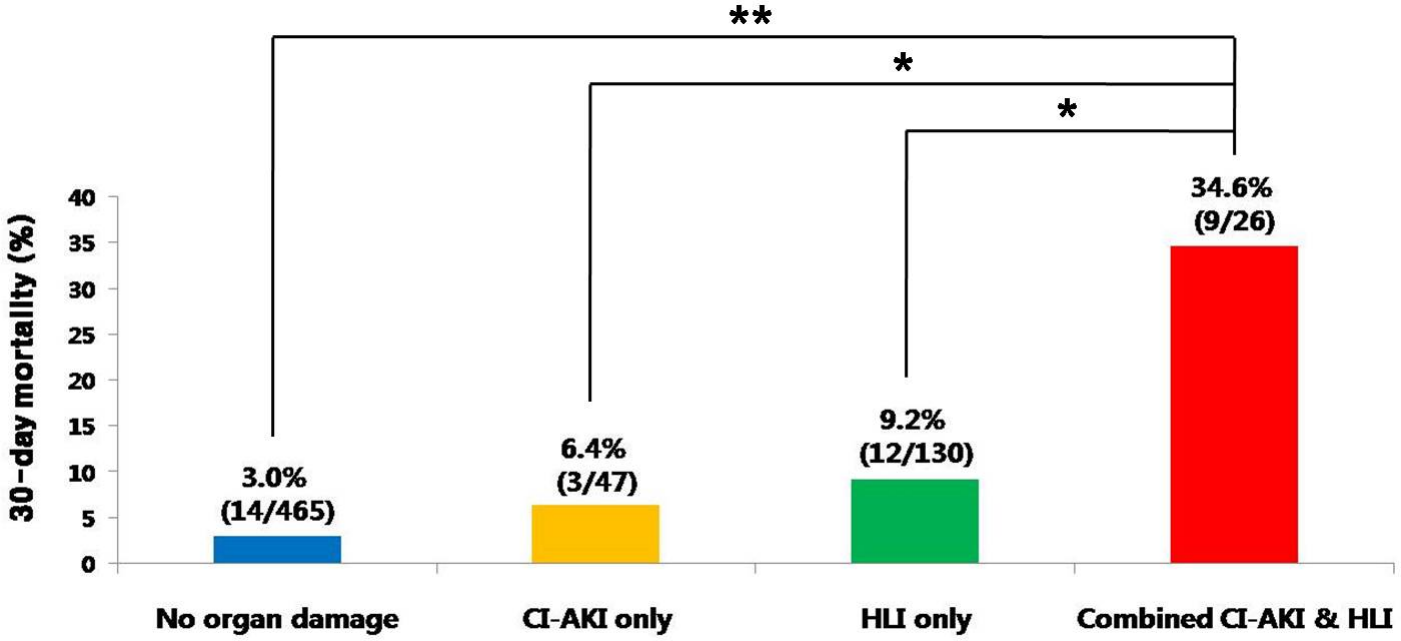
30-day mortality rates according to CI-AKI and HLI states. Abbreviations. CI-AKI = contrast induced acute kidney injury; HLI = hypoxic liver injury. ** p<0.0001, * p<0.01 by Fisher’s exact test with adjustments by permutation resampling.

The overall 30-day mortality of the study population was 5.7% (38/668) (Table 2). The 30-day mortality rates according to CI-AKI and HLI states were significantly different (p<0.001 by Fisher’s exact test; Fig 1). The 30-day mortality rate in the combined CI-AKI and HLI group (34.6%) was significantly higher than that in each group of the no organ damage (3.0%; p<0.0001), CI-AKI only (6.4%; p = 0.002), and HLI only (9.2%; p = 0.001), respectively. Although 36 patients (8.1%) had Killip class 4 during their initial presentation in the no organ damage group, only 14 patients (3.0%) died in this group. In contrast, although only 3 patients (12.0%) presented with Killip class 4 in the combined CI-AKI and HLI group, 9 patients (34.6%) in this group died.

**Table 2:** Prognostic outcome of the INTERSTELLAR cohort according to CI-AKI and HLI states.

	Total (n = 668)	No organ damage (n = 465)	CI-AKI only (n = 47)	HLI only (n = 130)	Combined CI-AKI and HLI (n = 26)
30-day mortality	38 (5.7%)	14 (3.0%)	3 (6.4%)	12 (9.2%)	9 (34.6%)
Composite MACCE	94 (14.1%)	45 (9.7%)	10 (21.3%)	24 (18.5%)	15 (57.7%)
All-cause mortality	66 (9.9%)	24 (5.2%)	10 (21.3%)	19 (14.6%)	13 (50.0%)
Non-fatal MI	15 (2.2%)	13 (2.8%)	0 (0%)	2 (1.5%)	0 (0%)
Non-fatal stroke	7 (1.0%)	3 (0.6%)	0 (0%)	3 (2.3%)	1 (3.8%)
TLR/TVR	6 (0.9%)	5 (1.1%)	0 (0%)	0 (0%)	1 (3.8%)

Data are expressed as numbers (%). Abbreviations. MACCE = major adverse cardiovascular and cerebrovascular event; MI = myocardial infarction; TLR = target lesion revascularization; TVR = target vessel revascularization; CI-AKI = contrast induced acute kidney injury; HLI = hypoxic liver injury.

### Long term prognosis according to CI-AKI and HLI states

**Fig 2:**
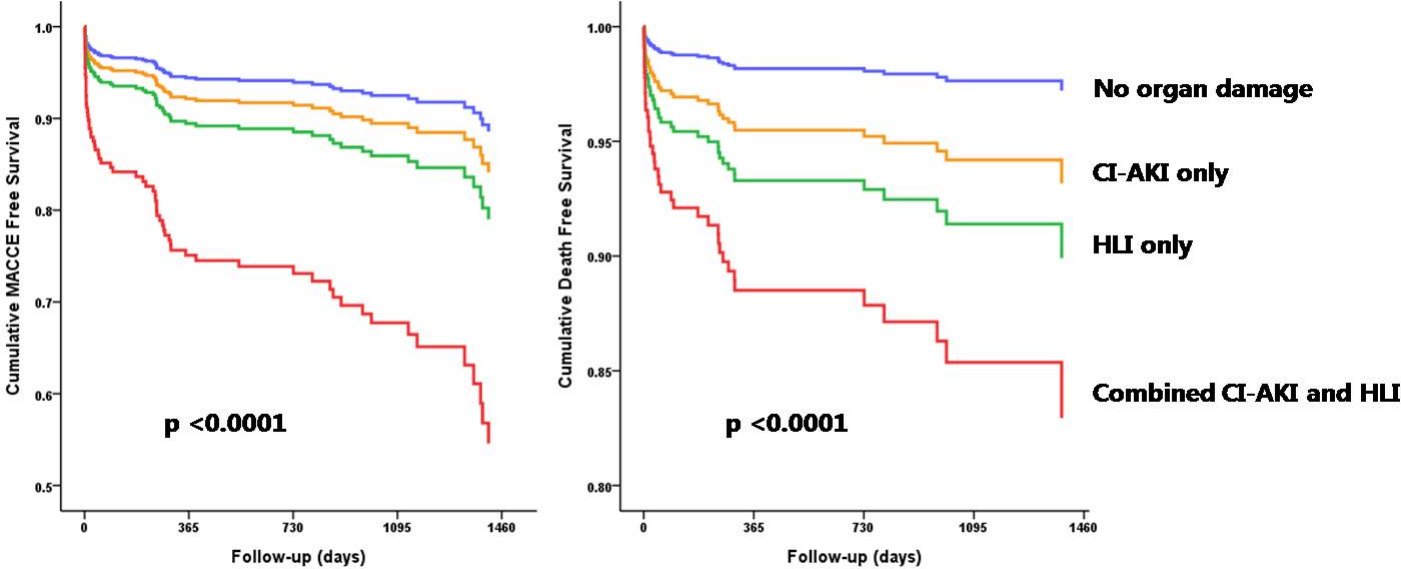
Survival probability plots of MACCE and all cause mortality according to CI-AKI and HLI state after adjustment for age, diabetes mellitus, hypertension, MVD, Killip class, and LVEF using Kaplan-Meier method. Abbreviations MACCE = major adverse cardiovascular and cerebrovascular event; CI-AKI = contrast induced acute kidney injury; HLI = hypoxic liver injury; MVD = multi-vessel disease; LVEF = left ventricular ejection fraction.

Over a mean follow-up period of 2.2±1.6 years, 94 MACCEs occurred (66 all-cause mortality, 15 non-fatal MI, 7 non-fatal stroke, and 6 ischemia-driven TLR/TVR) with an event rate of 14.1% (Table 2). The rate of MACCE and all-cause mortality were 9.7% and 5.2%, respectively, in the no organ damage group; 21.3% and 21.3%, respectively, in the CI-AKI only group; 18.5% and 14.6%, respectively, in the HLI only group; and 57.7% and 50.0%, respectively, in the combined CI-AKI and HLI group (Table 2). Consequently, Kaplan-Meier survival analysis for MACCE and all-cause mortality revealed that a combination of CI-AKI and HLI was associated with worst clinical outcomes (log rank p-value<0.0001 for both). In the multiple Cox proportional hazards analysis for MACCE, hypertension (HR 1.800, 95% CI 1.054–3.074, p = 0.031), LVEF (HR 0.954, 95% CI 0.932–0.976, p<0.0001), and combined CI-AKI and HLI (HR 4.996, 95% CI 2.309–10.811, p<0.0001) were significant risk factors (Table 3). In the multiple Cox proportional hazards analysis for all-cause mortality, age (HR 1.035, 95% CI 1.009–1.061, p = 0.008), diabetes mellitus (HR 2.145, 95% CI 1.126–4.087, p = 0.020), Killip class 4 (HR 2.517, 95% CI 1.113–5.692, p = 0.027), LVEF (HR 0.927, 95% CI 0.898–0.957, p<0.0001), and combined CI-AKI and HLI (HR 6.617, 95% CI 2.500–17.515, p<0.0001) were significant risk factors (Table 4). Furthermore, the survival probability plots of overall MACCE and all-cause mortality revealed that combined CI-AKI and HLI is associated with the worst prognosis, even after adjustment for age, diabetes mellitus, hypertension, MVD, Killip class, and LVEF (p<0.0001 for both) (Fig 2).

**Table 3:** Cox proportional hazards regression analysis for MACCE.

Variable	Simple			Multiple		
	HR	95% CI	p-value	HR	95% CI	p-value
Age	1.041	1.025–1.058	<0.0001	1.013	0.994–1.033	0.181
Male gender	0.788	0.498–1.247	0.309			
Diabetes	1.726	1.134–2.626	0.011	1.459	0.869–2.450	0.153
Hypertension	2.355	1.518–3.656	<0.0001	1.800	1.054–3.074	0.031
Dyslipidemia	1.259	0.795–1.994	0.326			
Multi-vessel disease	1.765	1.133–2.750	0.012	1.237	0.724–2.113	0.437
Killip class 4	3.874	2.328–6.447	<0.0001	1.692	0.789–3.627	0.177
Heart rate	1.015	1.007–1.023	<0.0001			
LVEF	0.940	0.921–0.960	<0.0001	0.954	0.932–0.976	<0.0001
CI-AKI	3.558	2.247–5.634	<0.0001			
HLI	2.445	1.617–3.697	<0.0001			
Combined CI-AKI & HLI	8.995	4.989–16.219	<0.0001	4.996	2.309–10.811	<0.0001

Abbreviations. CI = confidence interval; CI-AKI = contrast induced acute kidney injury; HLI = hypoxic liver injury; HR = hazard ratio; LVEF = left ventricular ejection fraction; MACCE = major adverse cardiovascular and cerebrovascular event; MI = myocardial infarction; TLR = target lesion revascularization; TVR = target vessel revascularization.

**Table 4:** Cox proportional hazards regression analysis for all-cause mortality.

Variable	Simple			Multiple		
	HR	95% CI	p-value	HR	95% CI	p-value
Age	1.065	1.043–1.086	<0.0001	1.035	1.009–1.061	0.008
Male gender	0.645	0.381–1.092	0.103			
Diabetes	2.211	1.355–3.607	0.001	2.145	1.126–4.087	0.020
Hypertension	2.690	1.561–4.634	<0.0001	1.695	0.838–3.428	0.142
Dyslipidemia	1.257	0.730–2.167	0.410			
Multi-vessel disease	2.103	1.209–3.658	0.008	1.077	0.522–2.220	0.841
Killip class 4	6.193	3.590–10.683	<0.0001	2.517	1.113–5.692	0.027
Heart rate	1.017	1.008–1.026	<0.0001			
LVEF	0.911	0.889–0.935	<0.0001	0.927	0.898–0.957	<0.0001
CI-AKI	5.085	3.055–8.463	<0.0001			
HLI	3.193	1.962–5.197	<0.0001			
Combined CI-AKI & HLI	13.407	6.793–26.460	<0.0001	6.617	2.500–17.515	<0.0001

Abbreviations. CI = confidence interval; CI-AKI = contrast induced acute kidney injury; HLI = hypoxic liver injury; HR = hazard ratio; LVEF = left ventricular ejection fraction; MACCE = major adverse cardiovascular and cerebrovascular event; MI = myocardial infarction; TLR = target lesion revascularization; TVR = target vessel revascularization.

## Discussion

### Incidence, predictors and cardiovascular risk of CI-AKI in patients with STEMI

A previous study had revealed that contrast volume, old age, impaired renal function, prior history of cardiovascular disease, acute coronary syndrome, diabetes mellitus, cardiogenic shock, anemia, and intra-aortic balloon pump support were independent predictors of CI-AKI, with 7% of CI-AKI incidence among CAD patients who had undergone PCI [8]. Moreover, they documented that CI-AKI was strongly associated with STEMI, chronic kidney disease, and cardiogenic shock–all of which may lead to worse outcome [8]. Another study reported that contrast volume/estimated glomerular filtration rate ratio (CV/eGFR) of>2.5, age, left ventricular dysfunction, and troponin level were independent predictors of CI-AKI in STEMI patients who have undergone primary PCI, with a 5.3% CI-AKI incidence [17]. A recent study revealed that contrast volume, white blood cell count, left anterior descending infarct-related artery, age, anemia, creatinine clearance of <60mL/min, and history of congestive heart failure were predictors of CI-AKI in STEMI patients who underwent primary PCI, with 16% CI-AKI incidence [7]. In concordance with these reports, our study showed that the CI-AKI group had higher initial creatinine levels and higher incidence of Killip class 4 compared to the no organ damage group, with 11% of CI-AKI incidence.

### Prognostic value of CI-AKI in patients with STEMI

Previous studies have revealed that CI-AKI is associated with worse prognosis, implying the detrimental effects of adscititious vital organ damage among patients with STEMI [6–9]. Since baseline renal function is a risk factor for CI-AKI development, a previous study had reported that baseline renal insufficiency is associated with markedly increased risk of both the 30-day and 1-year mortality among STEMI patients who had undergone primary PCI [18]. Another study reported that CI-AKI frequently complicates primary PCI, even among patients with normal renal function. Furthermore, it is associated with higher in-hospital mortality rate among patients with STEMI [19]. More recently, a large scale HORIZONS-AMI substudy demonstrated the prognostic significance of CI-AKI in both the short and long term outcomes among STEMI patients who had undergone primary PCI [7]. This study revealed that cardiac death, non-fatal MI, ischemic TVR occurred more frequently in the CI-AKI group than in the no CI-AKI group, in terms of both the 30-day and 3-year outcomes [7]. In concordance with these reports, our study revealed that CI-AKI was associated with worse outcome, with regards to both the short- and long-term prognoses.

### Pathophysiology of hypoxic hepatitis and its implication

The liver is a robust vital organ that is very sensitive to hemodynamic changes owing to its complex vascular system and high metabolic activity [20]. Although it has its own compensatory mechanism for disrupted blood flow (by extracting more oxygen from blood), this mechanism may become overwhelmed, and liver injury eventually occurs when circulatory failure persists [10,20,21]. Therefore, hypoxic hepatitis may be another adscititious vital organ damage in patients with STEMI, which affects the prognosis. Hypoxic hepatitis is diagnosed according to the following criteria: (1) cardiac, circulatory, and/or pulmonary failure; (2) severe and transient increase in transaminase levels; and (3) exclusion of other possible causes of liver damage [10,20]. Hypoxic hepatitis is defined as a peak transaminase level increase of more than 20-fold of the upper normal limit, if there is no histopathological validation [22,23]. However, previous studies suggested that the histopathological findings of hypoxic hepatitis may be present even below this 20-fold cut-off value of serum transaminase levels [24,25]. Another study reported 22% incidence of hypoxic hepatitis among intensive care unit patients with decreased cardiac output [26]. Furthermore, a separate study revealed that when hypoxic hepatitis takes place among intensive care unit patients, the mortality rate exceeds 50% [22]. Interestingly, although the mortality rate is high in these patients, the cause of death is usually due to the underlying disease, not hypoxic hepatitis *per se*.

### Prognostic value of HLI among patients with STEMI

Recently, we have proposed the prognostic impact of HLI in the emergency room setting among patients with STEMI [10]. We revealed that a 2-fold increase of serum transaminase level on admission predicts the outcome among STEMI patients who underwent primary PCI. In addition, we demonstrated that HLI is associated with post-PCI left ventricular dysfunction, which recovered after the acute phase of STEMI [10]. This result implies that STEMI patients with mild to moderate elevation of serum transaminase levels on admission are at higher risk of developing hypoxic hepatitis, of which the outcome may be fatal. In accordance to the previous study, we have adopted the definition of HLI on admission as an early marker of liver damage in the current study. With regards to the long-term prognosis, HLI was associated with worse outcome compared to that in patients with no organ damage. Several factors may contribute to the poor prognostic value of HLI on admission among STEMI patients who underwent primary PCI. First, HLI on admission may be an early marker of hypoxic hepatitis development. Since hypoxic hepatitis occurs as a consequence of severe left and/or right ventricular dysfunction, HLI on admission may imply extensive myocardial damage among patients with STEMI. Second, HLI on admission may signify sustained period for revascularization after the infarct-related coronary artery occlusion takes place, since transaminase elevation occurs several hours after STEMI occurrence. It may indicate increased irreversible myocardial damage burden leading to extensive myocardial necrosis.

### Prognostic impact of combined CI-AKI and HLI among patients with STEMI

The prognostic value of combined CI-AKI and HLI among STEMI patients who underwent primary PCI has not been demonstrated before. To the best of our knowledge, this was the first study that documented the prognostic impact of combined CI-AKI and HLI in interventionally-treated patients with STEMI. In our study, the 30-day mortality in the CI-AKI only group was 2-fold higher than the no organ damage group. Furthermore, the 30-day mortality rate increased when CI-AKI and HLI occurred in combination, more than 10-fold higher than that in the no organ damage group. In addition, the long term prognostic impact of combined CI-AKI and HLI persists in STEMI patients who undergo primary PCI, demonstrating a 5-fold or higher risk for both MACCE and all-cause mortality. A previous study revealed that STEMI patients with Killip class 4 had worse prognosis compared to those with Killip class 1 to 3 [27]. Indeed, STEMI patients with cardiogenic shock may develop a combination of CI-AKI and HLI owing to the transient hypoxemia and hypoperfusion status of the kidney and liver. Therefore, there may be a dissent from our opinion that combined CI-AKI and HLI has momentous prognostic impact. However, only 3 patients (12%) in the combined CI-AKI and HLI group had cardiogenic shock during their initial presentation, whereas 30-day mortality occurred in 9 patients (35%); this implied the probability of additional informative value of combined CI-AKI and HLI as a prognosticator. Therefore, we propose that combined CI-AKI and HLI is associated with worse prognosis in STEMI patients who underwent primary PCI, beyond the value of Killip classification.

## Limitations

There are several limitations in this study. First, this was an observational study that consisted of a Korean population. Second, histopathological confirmation of hypoxic liver damage, evidenced by centrilobular necrosis of hepatocytes, was not performed. However, as anti-platelet agents, intravenous heparinization, and glycoprotein IIb/IIIa inhibitors are frequently used in the acute phase of STEMI, liver biopsy may not be feasible in this situation, as the risk of fatal bleeding complication may overweigh the benefit of histopathological confirmation.

## Conclusion

Combined CI-AKI after index procedure and HLI on admission is associated with poorer clinical outcomes in patients with STEMI who underwent primary PCI.

## Supporting Information

S1 Dataset Dataset of the study population. (SAV)
